# Adaptation of the Korean Version of the Personal Financial Wellness Scale

**DOI:** 10.1186/s41155-025-00355-0

**Published:** 2025-05-30

**Authors:** Yongseok Kim, Sunghwan Cho, Sokho Lee, Joseph Ahn

**Affiliations:** 1 Department of Social Welfare, The Catholic University of Korea, 43 Jibong-Ro, Bucheon, Gyeonggi-Do 14662 South Korea; 2 Graduate School of Social Welfare, The Catholic University of Korea, 43 Jibong-Ro, Bucheon, Gyeonggi-Do 14662 South Korea; 3 Graduate Department of Addiction Studies, The Catholic University of Korea, 43 Jibong-Ro, Bucheon, Gyeonggi-Do 14662 South Korea

**Keywords:** Financial well-being, Personal Financial Wellness scale, Korean version of the PFW scale, Adaptation

## Abstract

**Background:**

In South Korea, there are many situations that can threaten financial well-being, a factor that has been known to affect both mental and physical health. However, there has so far not been an instrument to measure an individual’s subjective financial well-being.

**Objective:**

This study aims to adapt the Korean version of the Personal Financial Wellness (PFW) scale.

**Methods:**

The Korean version of the PFW scale was evaluated with 2,044 adults who participated in an online survey in South Korea. Confirmatory factor analysis was conducted to examine the factor structure of the PFW scale. Its validity was assessed by examining factor loadings, average variances extracted (AVE), and conceptual reliability (CR). In addition, the relationships between the PFW scale and the scales measuring theoretically relevant constructs were analyzed. Reliability was examined using Cronbach’s alpha coefficient, the corrected item-to-total correlation, the inter-item correlation, and McDonald's omega.

**Results:**

The evaluation of the psychometric properties of the Korean version of the PFW scale indicates that it exhibits a single-factor structure, consistent with the English version. Factor loadings, AVE, and CR all exceeded the recommended thresholds. The model also demonstrated consistent fit indices regardless of the subsample. Invariance of both configural and factor loadings was established across gender, while invariance of configural, factor loadings, and intercepts was confirmed across regions. Additionally, the PFW scale, a subjective measure of financial well-being, is more strongly related to health-related variables than monthly income, which is an objective indicator of financial well-being. Internal consistency reliability for the Korean version of the PFW scale was found to be satisfactory.

**Conclusions:**

The adaptation of the Korean version of the PFW scale provides a valuable instrument for researchers and practitioners in South Korea, where an instrument measuring an individual’s subjective financial well-being does not exist.

## Introduction

An individual’s financial situation is one of the most important factors that may affect their overall quality of life. People experiencing financial hardship may have difficulties paying household bills, buying nutritious food, and obtaining the appropriate health services they need. These financial situations can be very stressful and may result in both physical and mental health problems. For example, among college students, financial hardship and worry about future economic security are positively correlated with depression and suicidal ideation (Y. Lee et al., 2017). Similarly, in a study using longitudinal survey data, financial distress was negatively associated with subsequent self-reported measures of physical and mental health (Bialowolski et al., 2021). Some individuals have even committed suicide due to their financial hardship (Holkar, 2019).

Because most people are generally concerned about their financial situation, which can cause various problems in their lives, it is crucial to study the issue of financial well-being. Financial well-being has been conceptualized as “a state of being wherein a person can fully meet current and ongoing financial obligations, can feel secure in his or her financial future, and is able to make choices that allow enjoyment of life” (Consumer Financial Protection Bureau, 2015, p. 8) or as “the perception of being able to sustain the current and anticipated desired living standard and financial freedom” (Brüggen et al., 2017, p. 229). However, there is no consensus on its definition.

Existing definitions of financial well-being can be clustered into three groups (Brüggen et al., 2017; Prawitz et al., 2006). The first group defines financial well-being as a concept with both objective and subjective dimensions; the second group defines it as an objective concept, and the third group defines the term as a subjective term. For example, household income and savings are considered objective indicators (Greninger et al., 1996), and perceived satisfaction with current financial situation and perceptions regarding debt status are seen as subjective indicators (J. Kim et al., 2003).

Among the three approaches to defining and measuring the concept of financial well-being, it has been argued that the subjective approach is more appropriate than the objective approach (Brüggen et al., 2017; Mahdzan et al., 2020; Prawitz et al., 2006). Income, an objective indicator of financial well-being, serves as an example of the superiority of the subjective approach over the objective approach. In particular, different individuals with the same level of income may perceive their financial well-being differently (Mahdzan et al., 2020; Prawitz et al., 2006) because individuals’ financial status, money values, and other situations differ. However, the objective approach only measures the facets of one’s financial condition and fails to consider an individual’s subjective assessment of their financial well-being (Prawitz et al., 2006). Indeed, it is the individual who can accurately assess their level of financial well-being. In summary, an individual’s perception of financial well-being is more important than objective indicators in defining and measuring the concept.

In recent years, the subjective measurement of financial well-being has been improved through the use of scales, which provide a better, more accurate, and more reliable assessment of an individual’s financial well-being (Kamaluddin et al., 2018). Several scales including the CFPG Financial Well-Being Scale (Consumer Financial Protection Bureau, 2015) and the Reported Financial Wellbeing Scale (Melbourne Institute, 2020) have been developed for this purpose. One of the most commonly used scales to measure an individual’s subjective financial well-being is the Personal Financial Wellness (PFW) scale, formerly known as the InCharge Financial Distress/Financial Well-Being scale. The PFW scale was developed to measure perceived financial well-being as a single factor (Prawitz et al., 2006). The scale items were developed following a Delphi study with professors and financial education experts, who identified the concepts relevant to the measurement of financial distress and financial well-being (Prawitz et al., 2006). The scale is composed of eight items. Four items measure an individual’s sense of the present status of their financial well-being, and the other four items measure their reaction to their current financial situation (Prawitz et al., 2006). The PFW scale has been identified as a valid and reliable scale in the general population of adults in the United States (Prawitz et al., 2006) and has been used by more than 180 practitioners and researchers from various fields (Personal Finance Employee Education Foundation, 2010, as cited in Kamaluddin et al., 2018, p. 111). Additionally, the scale has been adapted in six European countries (Germany, Italy, the Netherlands, Slovenia, Spain, and the United Kingdom), and Malaysia (Buabang et al., 2022; Kamaluddin et al., 2018). A South African study highlighting the role of personal financial wellness in employees’ overall well-being and job productivity also utilized the PFW scale (Fouché & Manyaapelo, 2020).

In South Korea, there are many situations that can decrease financial well-being and increase financial stress. For example, people in Korea may experience financial stress due to concerns about skyrocketing housing prices. Moreover, parents with children must pay high private education costs for their children, and older adults worry about income security after retirement. In this context, the adaptation of the PFW scale into Korean will be instrumental for advancing both research and practice applications in the field of financial well-being. However, the PFW scale has not been adapted in the general population of adults in South Korea. The adaptation of the Korean version of the PFW scale would have a couple of advantages. First, it allows for the assessment of financial well-being among Korean adults, and second, it enables comparisons with adults in other countries.

### Purpose

This study aims to adapt the PFW scale for the general adult population in South Korea and it is the first to do so. This study addresses the following research question: Is the Personal Financial Wellness scale appropriate for use with Korean adults?

## Methods

### Study Sample

This study analyzed data from a national survey on gambling behavior. More specifically, a nationwide, general-population-based online survey was conducted in South Korea from July 1 to July 9, 2021, by a research firm to examine the impacts of coronavirus disease (COVID-19) on gambling behavior. The survey utilized a panel of 46,242 members who had consented to participate in research studies. From this panel, 8,245 members were identified as potential participants for this particular study, and 3,939 agreed to participate (participation agreement rate = 47.8%). However, only 2,044 individuals completed the survey, resulting in a response rate of 24.8%. To ensure the representativeness of the sample, this study employed a proportional quota sampling method-a non-probability sampling method in which specific quotas are set for subgroups within a population to match known population characteristics (Rubin & Babbie, 2024). Quotas were determined based on the 2021 Korean Census and were set for three key variables: age, gender, and region. The research firm monitored response counts in real time to ensure that recruitment continued until the predefined quotas for each subgroup were met. This approach enabled the final sample to closely approximate the national population distribution in terms of these demographics. Table 1 summarizes the demographic characteristics of the sample. Approval for this study was obtained from the Institutional Review Board of the authors’ university.

**Table 1:** *Sample demographics (N* = *2,044)*

Variable	*n*	%
Gender		
Male	1,043	51.0
Female	1,001	49.0
Age		
20 s	396	19.4
30 s	368	18.0
40 s	441	21.6
50 s	466	22.8
60 s	373	18.2
Years of education		
≤ 9	14	0.7
≤ 12	355	17.4
≤ 16	1,364	66.7
> 16	311	15.2
Region		
City	922	45.1
Province	1,122	54.9
Monthly personal income (in KRW)		
≤ 1,000,000	419	20.5
≤ 2,000,000	305	14.9
≤ 3,000,000	449	22.0
≤ 4,000,000	337	16.5
≤ 5,000,000	213	10.4
≤ 6,000,000	139	6.8
≤ 7,000,000	59	2.9
> 7,000,000	123	6.0
Monthly household income (in KRW)		
≤ 1,000,000	91	4.5
≤ 2,000,000	129	6.3
≤ 3,000,000	288	14.1
≤ 4,000,000	320	15.7
≤ 5,000,000	335	16.4
≤ 6,000,000	328	16.0
≤ 7,000,000	184	9.0
> 7,000,000	369	18.1

*Note*. The mean age of the participants was 44.36 years (*SD* = 13.59)

The exchange rate during data collection in July 2021 was approximately 1,100 KRW per USD. Accordingly, 1,000,000 KRW is equivalent to approximately $909 USD

### Measures and Translation

The translation process comprised the following steps. First, this study’s corresponding author translated the eight items of the scale into Korean. Then, a bilingual researcher majoring in social work conducted a back translation. Finally, a professor majoring in Korean-language education assured the accuracy and validity of the translation. Approval for developing the Korean version of the PFW scale into Korean was obtained from its developer via email communication (J. Hoffmier, personal communication, April 27, 2024). Each scale item is measured on a 10-point scale, and the possible total score of the scale ranges between 8 and 80, with a higher score indicating better perceived financial well-being.

Mental health was measured using the Kessler Psychological Distress Scale (K6) (Kessler et al., 2002). The K6 is a tool for measuring overall mental health and has been used in large-scale mental health epidemiologic studies in several countries, including the United States, where the tool was developed. The K6 consists of 6 questions with each item measured on a 5-point scale from 0 to 4 points, so the possible score range is 0 to 24. Higher scores on the K6 indicate more mental health problems. The Korean version of the K6 was evaluated using a Korean sample, demonstrating its ability to correctly identify 76% of individuals who has experienced psychiatric problems in the past year. The scale showed high internal consistency, with a Cronbach’s alpha of 0.91 (Kim, 2011). Physical health behaviors were measured using the two sub-scales of the Physical Health Behavior Scale (Yang & Kim, 2015), as health behavior is a known predictor of physical health (Dixon et al., 1993). The two sub-scales measure physical activities and the intake of healthy foods.

### Data analysis

A confirmatory factor analysis (CFA) was conducted to assess the factor structure of the Korean version of the PFW scale. Previous studies have found that the PFW scale has a single-factor structure, and the present study aim to confirm that the Korean version also exhibits this structure. The results of the CFA were compared with those of a previous study (Buabang et al., 2022), which adapted the PFW scale across seven countries. This prior study found that a modified one-factor model provided the best fit for the data, so the same model was tested in the present study using the statistical program R (version 4.4.2), and the ‘lavaan 0.6–19’ package (Rosseel, 2012). The R code for the modified one-factor model was obtained from the Open Science Framework (https://osf.io/gevmr/). Goodness of fit was evaluated using several indices, including chi-square, root mean square error of approximation (RMSEA), standardized root-mean-square residual (SRMR), comparative fit index (CFI), and Tucker–Lewis index (TLI). Recommended cut-off values are 0.95 or higher for CFI and TLI, and less than 0.08 for RMSEA (McNeish, 2023; Rezaee & Jafari, 2016).

Convergent validity of the Korean version was evaluated through factor loadings, average variance extracted (AVE), and construct reliability (CR) (Hair et al., 2018), with recommended thresholds of 0.50 or higher for factor loadings, 0.50 or higher for AVE, and 0.70 or higher for CR. Convergent validity was further supported by examining relationships with the K6 scale and the Physical Health Behavior Scale. To provide additional validity evidence, the strength of associations between the PFW scale and measures of mental health and physical health behaviors was compared to its associations with objective financial indicators (monthly personal and household income). A negative correlation was anticipated between the PFW scale and mental health and a positive correlation was expected between the PFW scale and physical health behaviors. Following the validity assessment, the internal consistency reliability of the Korean version was evaluated using Cronbach’s alpha coefficient, corrected item-to-total correlations, and inter-item correlations, and McDonald's omega (Hair et al., 2018; McNeish, 2018). SPSS 28.0 was used to analyze validity and reliability of the Korean version of the PFW scale.

## Results

### Item statistics

Descriptive statistics for the eight items of the Korean version of the PFW scale are presented in Table 2. Mean item scores ranged from 4.48 (*SD* = 1.67) for Item 3, which concerns feelings about one’s current financial condition, to 6.17 (*SD* = 2.14) for Item 6, which assesses the avoidance of leisure activities due to financial constraints. A couple of factors could explain the relatively high mean score for Item 6. First, the COVID-19 pandemic likely had a significant impact on respondents'leisure activities. During the pandemic, many outdoor activities were restricted or discouraged in Korea, which would have led to a general decrease in leisure time or changes in how people engage in these activities. Second, Korea's work culture may also play a role. Korea is known for having some of the longest working hours among OECD countries (OECD, 2023). The country’s work culture can lead to limited personal time and contribute to a culture where people often have to give up leisure activities due to work. The combination of these factors—COVID-19 restrictions and long working hours—could help explain why respondents report a higher frequency of giving up leisure activities. The greatest variability among items was observed for Item 5 (*M* = 5.82, *SD* = 2.52), which measures confidence in finding money to pay for financial emergencies. The relatively large standard deviation is likely due to the wide range of personal and household incomes in the sample. Income disparities may have contributed to the variation in responses, as personal monthly income and household income ranged from less than 1,000,000 KRW to over 7,000,000 KRW**.** The standard deviation for Item 5 was also the largest in a South African study (Fouché & Manyaapelo, 2020).

**Table 2:** Descriptive statistics of items

No	Item Text	*M* (*SD)*
1	What do you feel is the level of your financial stress today?	5.50 (1.94)
2	How satisfied are you with your present financial situation?	4.76 (1.85)
3	How do you feel about your current financial condition?	4.48 (1.67)
4	How often do you worry about being able to meet normal monthly living expenses?	5.16 (1.93)
5	How confident are you that you could find the money to pay for a financial emergency that costs about $1,000?	5.82 (2.52)
6	How often does this happen to you? You want to go out to eat, go to a movie, or do something else, but you don’t go because you can’t afford to	6.17 (2.14)
7	How frequently do you find yourself just getting by financially and living paycheck to paycheck?	6.15 (2.15)
8	How stressed do you feel about your personal finances in general?	5.54 (1.96)

### Confirmatory factor analysis

As a modified one-factor model with correlated errors has been shown to provide the best fit for the data in previous study (Buabang et al., 2022), the same model was explored in the present study. However, during the model modification process, the software encountered an identification issue, which resulted in the inability to compute standard errors. This suggests potential model misspecification or insufficient data to support the modified model.

Although the PFW scale consists of eight Likert-type items with a 10-point response format—data generally considered ordinal in nature (Li, 2016)—previous research, such as that by Buabang et al. (2022), has utilized maximum likelihood (ML) estimation. ML estimation rests on the assumptions of continuous variables and multivariate normality; which are often violated when analyzing ordinal data, potentially leading to biased parameter estimates (Li, 2016; Rhemtulla et al., 2012). Specifically, applying ML to ordinal data can result in inaccurate parameter estimates, standard errors, and chi-square statistics, especially when the number of categories is low and the distribution is asymmetrical (Beauducel & Herzberg, 2006; Johnson & Creech, 1983; Rhemtulla et al., 2012). Li (2016, p. 369) explicitly states that ML is “not, strictly speaking, appropriate for observed variables that are scaled ordinally.” In contrast, ULS—particularly when utilizing polychoric correlation matrices—is better suited for handling ordered categorical data, as it does not rely on the assumption of multivariate normality and provide more accurate parameter estimates (Forero et al., 2009; Li, 2016; Xia & Yang, 2019). Li’s (2016) simulation study demonstrated that ULS, along with diagonally weighted least squares (DWLS), which also uses a polychoric correlation matrix, yields more accurate and less biased estimates of factor loadings and interfactor correlations compared to ML when analyzing ordinal variables (p. 376, 383). This improved accuracy arises because ULS does not assume continuity and multivariate normality for the observed variables, which are often violated when Likert-scale data are treated as continuous. Therefore, the selection of ULS was intended to enhance the robustness and validity of the confirmatory factor analysis results for the Korean PFW scale.

Table 3 shows the model fit indices for the one-factor model of the Korean version of the PFW scale. The chi-square value was 292.48 with 20 degrees of freedom. The CFI was 0.99, the TLI was 0.99, the RMSEA was 0.08, and the SRMR was 0.06, with all indices meeting the recommended cut-off values. The large chi-square value is likely attributable to the substantial sample size (*N* = 2,044).

**Table 3:** Model fit indices for the one-factor model of the Korean version of the PFW scale

Model	χ2	df	CFI	TLI	RMSEA	SRMR
Korean version of the Personal Financial Wellness Scale	292.48	20.00	.99	.99	.08	.06

*Note*. χ^2^ = chi-square; *df* degrees of freedom, *CFI* Comparative fit index, *TLI* Tucker–Lewis index, *RMSEA* Root mean square error of approximation, *SRMR* Standardized root mean square residual

Additionally, random subsamples of 500, 1,000, and 1,500 individuals were drawn from the original study sample, and the results were subsequently compared to those obtained from the full original sample. The results shown in Table 4 indicated that the model fit indices across the subsamples did not differ significantly from those in the original study sample. The goodness-of-fit indices demonstrated consistent and adequate model fit regardless of the sample size.

**Table 4:** Model fit indices for randomly selected subsamples

Model	χ2	df	CFI	TLI	RMSEA	SRMR
N = 500	78.78	20	.99	.99	.08	.07
N = 1,000	131.22	20	.99	.99	.08	.06
N = 1,500	226.09	20	.99	.98	.08	.07
N = 2,044*	292.48	20	.99	.99	.08	.06

*Note*. N = sample size; χ^2^ = chi-square; *df* degrees of freedom, *CFI* Comparative fit index, *TLI* Tucker–Lewis index, *RMSEA* Root mean square error of approximation, *SRMR* Standardized root mean square residual

^*^ The original number of study samples

The model fit indices for the Korean version of the PFW scale were also compared to those reported in the multinational validation study by Buabang et al. (2022), as shown in Table 5. The chi-square value for the Korean version of the PFW scale was 292.48 with 20 degrees of freedom, while the seven international versions ranged from 11.82 to 37.06 with 8 degrees of freedom. The Korean PFW scale demonstrated strong fit indices (CFI = 0.99, TLI = 0.99), which are highly comparable to, and in some instances exceed, those reported for the modified one-factor model in the seven international versions (CFI range = 0.97 to 1.00; TLI range = 0.90 to 0.99; Buabang et al., 2022). While the RMSEA for the Korean version (0.08) was on the cusp of acceptability and slightly higher than most international versions (range = 0.04 to 0.13, with most ≤ 0.09), and the SRMR (0.06) was also slightly higher (international range = 0.01 to 0.03), both remained within acceptable thresholds for model fit, particularly given the much larger sample size and different degrees of freedom in the Korean validation. Although prior research suggested a modified one-factor model with 12 additional correlated errors, the present study found that the baseline model demonstrated satisfactory fit indices without any modifications. This parsimony strengthens the model's applicability in the Korean context.

**Table 5:** *Comparison of the model fit indices of the Korean version of the PFW scale with those reported in Buabang *et al*.’s study*

Country	*N*	χ2	*df*	CFI	TLI	RMSEA	SRMR
Korea	2,044	292.48	20.000	.99	.99	.08	.06
Germany	205	22.46	8.00	.99	.96	.09	.03
Italy	213	21.76	8.00	.99	.95	.09	.02
The Netherlands	207	37.06	8.00	.97	.90	.13	.03
Slovenia	212	13.62	8.00	1.00	.98	.06	.01
Spain	203	14.09	8.00	1.00	.98	.06	.02
UK	288	11.82	8.00	1.00	.99	.04	.01
US	222	12.55	8.00	1.00	.99	.05	.01

*Note.* *N* sample size, χ2 = chi-square, *df* degrees of freedom, *CFI* Comparative fit index, *TLI* Tucker–Lewis index, *RMSEA* Root mean square error of approximation, *SRMR* Standardized root mean square residual

### Measurement Invariance Test

Measurement invariance was examined by specifying a sequence of three nested models, each progressively constraining the factor structure, factor loadings, and intercepts to be equal across groups. Tables 6 and 7 present the results of the measurement invariance test across gender and region, respectively.

**Table 6:** Measurement invariance test for gender (male = 1,043, female = 1,001)

Invariance Model	χ2	df	CFI	TLI	RMSEA	SRMR	χ2 diff	df diff	p
Configural	305.39	40	.99	.99	.08	.06			
Factor Loading	310.46	47	.99	.99	.07	.06	5.16	7	.640
Intercept	489.53	110	.99	.99	.06	.07	179.07	63	<.001

*Note.* χ2 = chi-square; *df* degrees of freedom, *CFI* Comparative fit index, *TLI* Tucker–Lewis index, *RMSEA* Root mean square error of approximation, *SRMR* Standardized root mean square residual, *diff*. difference, *p* probability

**Table 7:** Measurement invariance test for region (city = 922, local province = 1,122)

Invariance Model	χ2	df	CFI	TLI	RMSEA	SRMR	χ2 diff	df diff	p
Configural	296.09	40	.99	.99	.08	.06			
Factor Loading	306.80	47	,99	,99	.07	.06	10.70	7	.152
Intercept	368.64	110	.99	.99	.05	.06	61.84	63	.518

*Note.* χ2 = chi-square; *df* degrees of freedom, *CFI* Comparative fit index, *TLI* Tucker–Lewis index, *RMSEA* Root mean square error of approximation, *SRMR* Standardized root mean square residual, *diff*. difference, *p* = probability

The results shown in Table 6 indicated that the factor structure (CFI = 0.99, TLI = 0.99, RMSEA = 0.08) and factor loadings (χ2 diff. = 5.16, *p* = 0.640) were invariant across gender, whereas the intercepts were not (χ2 diff. = 179.07, *p* < 0.001). Accordingly, latent mean comparisons across gender should be interpreted with caution. The results in Table 7 showed that configural (CFI = 0.99, TLI = 0.99, RMSEA = 0.08), factor loading (χ2 diff. = 10.70, *p* = 0.152), and intercept (χ2 diff. = 61.84, *p* = 0.518) invariances were supported for region, indicating relatively strong measurement invariance.

### Validity and Reliability

Table 8 presents the factor loadings of the Korean version of the PFW scale. All items loaded significantly on the single factor, with loadings ranging from 0.690 to 0.902. The AVE was 0.673, and the CR was 0.942,.indicating that the scale has good convergent validity.

**Table 8:** Factor Loadings of the Korean Version of the PFW Scale

Item No	Factor loading	AVE	CR
1	.816^***^	.673	.942
2	.790^***^		
3	.847^***^		
4	.897^***^		
5	.690^***^		
6	.731^***^		
7	.862^***^		
8	.902^***^		

*Note.* ^***^p <.001

As expected, the Korean version of the PFW scale was negatively correlated with mental health ($$r=-.531, p<.01$$) and positively correlated with physical activities ($$r=.231, p<.01)$$ and healthy food intake ($$r=.290, p<.01$$) (see Table 9). These findings collectively demonstrate that the Korean version of the PFW scale exhibits the expected relationships with the theoretically relevant constructs. Most notably, the correlations between the Korean version of the PFW scale and the health-related variables were stronger than those between monthly personal/household income and the health-related variables, as shown in Table 9. This result suggests that the PFW scale, a subjective measure of financial well-being, is more closely associated with health-related variables than objective measures of financial well-being, such as monthly income.

**Table 9:** Correlations between the Korean Version of the PFW Scale and other Measures

Measure	PFW scale	Personal income	Household income
Mental health	−.531^**^	-.128^**^	-.198^**^
Physical activities	.231^**^	.115^**^	.137^**^
Healthy food intake	.290^**^	.111^**^	.187^**^

*Note.* ^**^p <.001

The internal consistency reliability of the Korean version of the PFW scale was found to be satisfactory (see Table 10). Cronbach's alpha coefficient was 0.928 and McDonald’s omega was 0.934, both above the recommended lower limit of 0.70 (Hair et al., 2018; Roco-Videla et al., 2024). The item–total correlations ranged from 0.650 to 0.847, and the inter-item correlations ranged from 0.458 to 0.774.

**Table 10:** Internal Consistency Reliability Results

Item	Inter-item correlation								Item–total Correlation	Reliability
	1	2	3	4	5	6	7	8		
1	—								.752	Cronbach’s α =.928 McDonald’s ω =.934
2	.686	—							.733	
3	.710	.767	—						.770	
4	.707	.691	.749	—					.834	
5	.458	.472	.501	.578	—				.650	
6	.479	.474	.481	.596	.608	—			.701	
7	.619	.583	.604	.732	.636	.760	—		.824	
8	.774	.662	.699	.762	.557	.645	.769	—	.847	

## Discussion

This study aimed to adapt the PFW scale for the Korean population. The findings indicate that the Korean version of the PFW scale possesses strong psychometric properties for measuring subjective financial well-being. It maintains a single-factor structure, consistent with the English version. Most of the model fit indices for the one-factor model were acceptable for both the original sample and the subsamples. Compared to the findings from Buabang et al.’s (2022), the model fit for the Korean PFW scale was generally strong and largely comparable. However, such direct comparisons of model fit statistics should be interpreted cautiously due to inherent limitations, such as differences in sample characteristics, model specifications (degrees of freedom), and estimation methods across the studies. The scale’s convergent validity was supported by factor loadings, AVE, and CR that exceeded recommended thresholds. Further evidence of convergent validity was provided by significant correlations with theoretically related constructs, including mental health, physical activities, and healthy food intake. Notably, the PFW scale, a subjective measure of financial well-being, exhibited stronger associations with these health-related variables than did monthly income, an objective indicator of financial well-being. Multiple evaluations confirmed the scale’s overall internal consistency reliability of the scale as satisfactory.

The adaptation of the PFW scale significantly contributes to the measurement of financial well-being by providing a comprehensive subjective assessment tool. The findings of this study demonstrate its greater efficacy in explaining the multifaceted aspects of financial well-being compared to conventional objective indicators, such as monthly income, debt, and savings. Specifically, the PFW scale showed stronger correlations with health variables (i.e., mental health, physical activities, and healthy food intake) compared to monthly income, for both individuals and households. This suggests that subjective financial well-being, as measured by the scale, may be a more accurate predictor of health outcomes compared to objective measures of financial well-being. This aligns with existing literature, which asserts that subjective measures of financial well-being often better reflect the reality of a person's finances and how this influences their overall well-being (Brüggen et al., 2017; Mahdzan et al., 2020). Moreover, future research should examine the relationship between the PFW scale scores and objective financial indicators to further evaluate the relative utility of subjective versus objective measures in predicting important outcomes such as psychological well-being, health behaviors, and financial decision-making.

The Korean version of the PFW scale can be applied in a range of behavioral health and human service contexts. First, practitioners working with individuals experiencing chronic stress, addictive behaviors, or economic marginalization could use the scale to assess perceived financial strain and its psychosocial impact. Integrating the scale into assessments or program evaluations would provide a more holistic understanding of clients’ circumstances, particularly in settings addressing co-occurring mental health and financial stress. Second, the PFW scale is particularly relevant for understanding financial well-being among vulnerable populations. For example, in South Korea, where older adults often experience insufficient pension coverage, limited employment opportunities, and heightened economic insecurity (Kim & Kim, 2024; Ku et al., 2021), the scale could be valuable for assessing the financial well-being of older adults. Similarly, investigating the PFW scale scores among low-income populations could provide insights into the psychological impact of financial hardship and inform targeted interventions. Accordingly, future research should examine how PFW scale scores vary across different income levels, age groups, and socioeconomic statuses to identify populations at the greatest risk of financial distress. Third, although various financial assistance programs (e.g., basic livelihood security program, emergency welfare support program, national employment support system) have been implemented in South Korea in response to the growing economic crisis, systematic tools to evaluate their effectiveness—particularly in terms of improving individuals’ perceived financial well-being—remain underdeveloped. Traditional evaluations often focus on objective indicators such as income thresholds or employment rates, offering limited insight into whether such interventions actually alleviate financial distress from the perspective of beneficiaries. In this context, the PFW scale could fill this gap by enabling policymakers to assess whether support programs enhance not only material outcomes but also subjective financial security among vulnerable populations.

Despite its important contributions, this study has several methodological limitations. First, the fact that the data were collected during the COVID-19 pandemic may have influenced people’s financial perceptions in unusually ways, particularly due to short-term instability in labor market participation and household income streams. Although the sample was demographically representative, future research should evaluate the scale’s psychometric performance across population subgroups that may experience financial distress in distinct ways—such as irregularly employed individuals, single-person households, or persons in recovery from behavioral addictions. The application of stratified analytical techniques alongside measurement invariance testing would facilitate the evaluation of whether the scale operates equivalently across demographically and socioeconomically diverse groups. Second, longitudinal research assessing the stability of the PFW scale scores over time and their sensitivity to changing economic conditions would be valuable. This would provide empirical evidence regarding the scale’s responsiveness to changes associated with recovery and adaptation processes over time. Third, although the response rate of 24.8% is consistent with rates commonly observed in online survey research (Nulty, 2008), the possibility of non-response bias cannot be ruled out. It is possible that individuals who chose to participate differ in meaningful ways from those who did not, which may affect the generalizability of the findings. The final limitation concerns the evaluation of the convergent validity. Comparing the findings with those obtained using an established financial well-being scale, such as the one developed by Kamaluddin et al. (2018), would have strengthened the evaluation of convergent validity. However, the current study did not include other financial well-being measures in the survey questionnaire, making such an analysis unfeasible. Future studies are encouraged to enhance the convergent validity of the Korean version of the PFW scale by examining its relationships with other established scales.

## Conclusion

This study is the first to adapt the Korean version of the Personal Financial Wellness scale, making a valuable contribution to the measurement of subjective financial well-being in the Korean context. Its strong psychometric properties and ability to comprehensively assess perceived financial well-being make it useful for both research and practical applications. The overall model fit was generally consistent with findings from previous multinational studies, supporting the scale’s potential for cross-cultural generalizability. To build on this research, future studies should focus on comparing subjective and objective measures of financial well-being, examining variations across different demographic and socioeconomic groups, and investigating longitudinal relationships with health and well-being outcomes. These studies will deepen our understanding of financial well-being and its implications for individual and societal outcomes in South Korea.

## Data Availability

This study dataset is available upon request and with completion of the data user agreement.

## Abbreviations

PFW: Personal financial wellness
COVID-19: Coronavirus disease
KRW: Korean won
USD: United States dollar
CFA: Confirmatory factor analysis
RMSEA: Root mean square error of approximation
SRMR: Standardized root-mean-square residual
CFI: Comparative fit index
TLI: Tucker–Lewis index
AVE: Average variance extracted
CR: Construct reliability
ML: Maximum likelihood
ULS: Unweighted least squares
